# Role of aIF1 in *Pyrococcus abyssi* translation initiation

**DOI:** 10.1093/nar/gky850

**Published:** 2018-09-20

**Authors:** Auriane Monestier, Christine Lazennec-Schurdevin, Pierre-Damien Coureux, Yves Mechulam, Emmanuelle Schmitt

**Affiliations:** Laboratoire de Biochimie, Ecole polytechnique, CNRS, Université Paris-Saclay, 91128 Palaiseau cedex, France

## Abstract

In archaeal translation initiation, a preinitiation complex (PIC) made up of aIF1, aIF1A, the ternary complex (TC, e/aIF2-GTP-Met-tRNA_i_^Met^) and mRNA bound to the small ribosomal subunit is responsible for start codon selection. Many archaeal mRNAs contain a Shine-Dalgarno (SD) sequence allowing the PIC to be prepositioned in the vicinity of the start codon. Nevertheless, cryo-EM studies have suggested local scanning to definitely establish base pairing of the start codon with the tRNA anticodon. Here, using fluorescence anisotropy, we show that aIF1 and mRNA have synergistic binding to the *Pyrococcus abyssi* 30S. Stability of 30S:mRNA:aIF1 strongly depends on the SD sequence. Further, toeprinting experiments show that aIF1-containing PICs display a dynamic conformation with the tRNA not firmly accommodated in the P site. AIF1-induced destabilization of the PIC is favorable for proofreading erroneous initiation complexes. After aIF1 departure, the stability of the PIC increases reflecting initiator tRNA fully base-paired to the start codon. Altogether, our data support the idea that some of the main events governing start codon selection in eukaryotes and archaea occur within a common structural and functional core. However, idiosyncratic features in loop 1 sequence involved in 30S:mRNA binding suggest adjustments of e/aIF1 functioning in the two domains.

## INTRODUCTION

Eukaryotic translation initiation involves a 43S preinitiation complex (PIC) consisting of the initiator methionyl-tRNA:eIF2:GTP ternary complex (TC) bound to the small ribosomal subunit in the presence of initiation factors (eIFs) 1, 1A, 5 and 3. For most mRNAs, discovery of the start codon occurs upon scanning of the mRNA by the 43S PIC until an AUG codon in a correct nucleotidic context is found (1). Recognition of the start codon triggers eIF1 departure. This further provokes the release of the Pi group arising from GTP hydrolysis on eIF2 and then the departures of eIF2-GDP, eIF5 and eIF3 (2). Finally, an elongation-proficient 80S complex is formed through large subunit joining with the help of eIF5B and eIF1A (3,4). eIF1 was shown to play a central function in translation initiation. It promotes TC binding to the ribosome (5,6), is necessary for mRNA scanning and essential to the accuracy of start codon selection by preventing translation initiation at non-AUG codons or at AUG codons in a non-optimal nucleotidic context (7–11). eIF1 function was proposed to be associated with two distinct conformations of the ribosome, an ‘open’ scanning competent conformation and a ‘closed’ state unable to scan mRNA, only reached when a correct AUG codon has been found (8,12). Consistent with this idea, it was shown that final tRNA accommodation within the P site after start codon recognition was triggered by eIF1 departure (13). Importantly, structural data on 40S:eIF1 complexes have identified the binding site of eIF1 on the small ribosomal subunit, in front of the P tRNA binding site (14,15). A predicted steric clash between the P site tRNA and a basic loop of eIF1 was in agreement with the requirement of eIF1 release for full accommodation of the tRNA. Finally, recent cryo-EM studies of eukaryotic translation initiation complexes have highlighted some of the structural changes that lead to a closed state of the PIC with the initiator tRNA base-paired to the start codon (16–19). These structures have shown how accommodation of the initiator tRNA at the P site, from a P_OUT_ to a P_IN_ position (20) led to a closure of the mRNA channel in the 40S subunit, destabilizing eIF1 and provoking its departure.

In archaea, the pre-initiation complex consists of the initiator methionyl-tRNA:aIF2:GTP ternary complex (TC) bound to the small ribosomal 30S subunit in the presence of the mRNA and of two other initiation factors, aIF1 and aIF1A. aIF1, 1A and 2 are homologous to their eukaryotic counterparts (21–23). This led to the idea that eukaryotic and archaeal translation initiations have in common a ribosomal core complex containing the TC bound to the small ribosomal subunit in the presence of the mRNA, e/aIF1 and e/aIF1A. Consistent with this view, recent cryo-EM structures of archaeal *Pyrococcus abyssi* PIC showed some similarities with eukaryotic PICs. In particular, binding sites of aIF1 and aIF1A on the small ribosomal subunit are conserved in both domains of life (24). Moreover, in the *Sulfolobus solfataricus* system, aIF1 promotes TC binding to the ribosome, favors discrimination against non-canonical start codons and enhances translation efficiency (25,26). In contrast to the eukaryotic case, long-range scanning does not occur in archaea because of the presence of Shine-Dalgarno sequences or of very short 5′UTR. Moreover, the eIF3 and eIF5 factors are absent. Finally, a state of the PIC with tRNA at a remote position from the P site was identified in the archaeal case (24). Thus, even if translation initiation in eukaryotes and archaea occurs within a similar ribosomal core complex, to some extent, the molecular functions of the three initiation factors may have evolved during the differentiation of the eukaryotic and archaea branches. However, few experimental data concerning archaeal initiation are available to date (e.g. (24–30)).

In the present work, we performed structure function studies of archaeal aIF1 in translation initiation. From the crystal structure of aIF1 from *Methanocaldococcus jannaschii* and sequence alignments, we highlighted archaeal specificities of the factor. In particular, we showed that the N-terminal mobile domain contains a zinc binding module in >60% of archaeal aIF1 sequences. Using fluorescence anisotropy, we showed that bindings of mRNA and *P. abyssi* aIF1 (Pa-aIF1) to the small ribosomal subunit (Pa-30S) are synergistically coupled. Binding of Pa-aIF1 involves residues of the basic loop 1 that are devoted either to the binding to the 30S or to the stabilization of the 30S:mRNA complex. Using primer extension inhibition assays, we identified toeprinting patterns characteristic of PICs obtained with various IF combinations. We observe that aIF1 favors a dynamic conformation of the PIC, reflecting a non-fully accommodated initiator P site tRNA. Such a conformation prevents wrong PICs to go further in the initiation pathway. Overall the data gives further support to the idea that even if long-range scanning of the mRNA by the PIC does not occur in archaea because of the presence of the SD sequence, there is a local scanning for the precise positioning of the initiator tRNA to the start codon (24). By favoring the dynamic conformation of the PIC, aIF1 would promote this local scanning as observed in the IC1-P_IN_ cryo-EM structure (24).

## MATERIALS AND METHODS

### Bacterial strains, plasmids and site-directed mutagenesis

*Escherichia coli* XL1-Blue and BL21-Rosetta (Invitrogen) strains have been used for cloning and expression experiments. The gene encoding aIF1 from *P. abyssi* was amplified from genomic DNA and cloned into pET3alpa plasmid to produce a tagged-free version of the factor starting with the MVP sequence. The QuickChange method was used to generate mutations (31). Unlabeled Pa-aIF1* corresponds to a variant of Pa-aIF1 carrying the mutations C62S and K45C. Variants R31A, Y32A, K34A have been created in the context of wild-type protein Pa-aIF1 for toeprinting experiments and in the context of Pa-aIF1* for fluorescence anisotropy measurements. The genes encoding the three subunits of Pa-aIF2 and Pa-aIF1A have been cloned in pET3alpa vectors as described in (32) and (24) to produce tagged-free versions of the factors.

### Purification of Pa-aIF1 and its variants

Typically, 500 ml cultures of BL21-Rosetta cells overproducing Pa-aIF1 were harvested, resuspended in 20 ml of buffer A (10 mM HEPES pH 7.5, 3 mM 2-mercaptoethanol, 0.1 mM EDTA, 0.1 mM PMSF, 0.1 mM benzamidine) plus 100 mM NaCl and disrupted by sonication. The crude extract was then heated 10 min at 70°C. After pelleting the non-thermostable proteins, the supernatant was loaded onto a Q-Hiload column (10 mm × 4 cm; GE Healthcare) equilibrated in buffer A plus 100 mM NaCl. The flow through was recovered and loaded onto an S-Hiload column (10 mm × 4 cm; GE Healthcare) equilibrated in buffer A plus 100 mM NaCl. Pa-aIF1 was eluted by applying a NaCl gradient (100–600 mM). The recovered sample was then concentrated and loaded onto a superdex 75 (HR 10/30, GE Healthcare). The purified protein was finally concentrated to ∼200 μM and stored at –20°C in buffer A with 55% glycerol for further use in toeprinting experiments (Supplementary Figure S1). Pa-aIF1 variants were purified using the same protocol adapted to the isoelectric points of the proteins and to their behaviors on the ion exchange columns.

### Purification of Pa-30S, Pa-aIF1A and Pa-aIF2

Purification of Pa-30S and Pa-aIF1A were performed as described in (24). The untagged version of Pa-aIF2 was purified as follows. Cultures (250 ml) of cells overproducing each of the three subunits of Pa-aIF2 (32) were harvested, mixed in 40 ml of buffer B (500 mM NaCl, 10 mM HEPES pH 7.5, 10 mM 2-mercaptoethanol, 0.1 mM EDTA, 0.1 mM PMSF, 0.1 mM benzamidine) and disrupted by sonication. After centrifugation, the supernatant was heated for 10 min at 80°C. After pelleting, the supernatant was loaded onto a Q-Hiload column (10 mm × 4 cm; GE Healthcare) equilibrated in buffer B. The flow through was recovered and diluted two fold with buffer A. The sample was then loaded onto an S-Hiload column (10 mm × 4 cm; GE Healthcare) equilibrated in buffer A plus 250 mM NaCl. The assembled heterotrimer was eluted by applying a step of buffer A plus 700 mM NaCl. The recovered sample was then concentrated to 1 ml. The final heterotrimer preparation was obtained after purification by molecular sieving on a Superdex 200 HR column (10 mm × 30 cm, GE Healthcare) equilibrated in buffer B. Finally, glycerol (55% final concentration) and 1 mM Gpp(NH)p-Mg^2+^ were added. The protein was stored at –20°C for further use in toeprinting experiments. Routinely, 6 mg of purified protein were obtained from the three mixed 250 ml cultures.

### Crystal structure of aIF1 from *Methanocaldococcus jannaschii*

The gene encoding aIF1 from *M. jannaschii* (Mj-aIF1) was amplified from genomic DNA and cloned into pET15blpa to produce an N-terminally his-tagged version of the factor. A culture (500 ml) of cells overproducing Mj-aIF1 was harvested, resuspended in 20 ml of buffer B and disrupted by sonication. After centrifugation, the crude extract was loaded onto a Talon affinity column (Clontech) and the protein was eluted with buffer B containing 125 mM imidazole. 0.25 unit of thrombin per mg of Mj-aIF1 was added. The sample was then dialyzed in buffer A containing 10mM CaCl_2_ overnight at 4°C. The detagged protein was further purified by passing through a Talon affinity resin and by ion exchange chromatography on an S-Hiload column and finally dialyzed against buffer A. After concentration to 3 mg/ml, the protein was used for crystallization experiments (Supplementary Figure S1).

Crystals of Mj-aIF1 were obtained by the hanging drop method in 25% PEG 3350, 0.1 M HEPES pH 7.5. Before data collection, crystals were cryo-protected by soaking in a solution containing 25% PEG3350, 0.1 M HEPES pH 7.5, 10% glycerol. Data were collected at the SOLEIL Proxima-1 beamline and processed using XDS (33) and programs of the CCP4 package (34). The structure was solved by single isomorphous replacement and anomalous scattering (SIRAS) using a platinum derivative (one hour soaking in 2 mM K_2_PtCl_4_) collected at the peak absorption wavelength (1.0716 Å) and the SOLVE program (35). After solvent flattening using RESOLVE (36), the model was manually built in Coot (37). Refinement was performed using PHENIX standard procedures (38). Statistics are shown in Supplementary Table S1.

### Atomic absorption spectroscopy

Before measurements, untagged Mj-aIF1 and Pa-aIF1 were dialyzed overnight at 4°C against a buffer containing 10 mM HEPES pH 7.5, 150 mM KCl, 3 mM 2-mercaptoethanol and 0.1 mM EDTA. Standard solutions containing various zinc concentrations from 0.625 to 10 μM were prepared by dilution in the dialysis buffer of a 15.3 mM ZnCl_2_ standard solution (Merck). Zinc atomic absorbencies were measured at 213.9 nm in the peak height mode during 5 s after 0.1 ml injections using a Varian AA775 spectrophotometer equipped with an air-acetylene burner. Reported value is the mean ± SD from two independent experiments (Supplementary Figure S1C).

### Preparation of methionylated tRNAs

tRNA_f_^Met^ A_1_-U_72_ was produced in PalΔmetZWVΔmetY::kan *E. coli* strain from PBSTNAV-tRNA_f_^Met^A_1_-U_72_, purified and aminoacylated as described (39,40). tRNA_i_^Met^, tRNA_m_^Met^ and tRNA _m_^Met^ 3G3C were produced in JM101Tr from cloned versions of the tRNAs (30,41), purified and aminoacylated as described (39).

### Fluorescent labeling of Pa-aIF1 and anisotropy measurements

We designed a mutated aIF1 protein into which C62 was mutated into S and K45 was mutated into C (see Results). The resulting protein (pa-aIF1-C62S-K45C) was labeled using a 5- to 15-fold higher molar concentration of MDCC (7-diethylamino-3-((((2-maleimidyl)ethyl)amino)carbonyl)coumarin, Fluka) during 2 h at 30°C. After reaction, the labeled protein was purified from unreacted MDCC using molecular sieve or ion exchange chromatography, as described above, depending on the isoelectric point of the protein. Usually, Pa-aIF1* and the variants were labeled with a yield ∼50%. The labeled protein was stored at –80°C at a concentration of nearly 100 μM.

For anisotropy measurements, Pa-aIF1* was diluted at the desired concentration to a final volume of 220 μl in a buffer containing 10 mM HEPES pH 7.5, 200 mM NaCl, 10 mM MgAc and 0.1% Tween 20. All measurements have been performed in a Hellman 1 cm × 0.4 cm cuve at 51°C with a FP-8300 JASCO spectrofluorometer, except titration of Pa-aIF1* with 30S:mRNA (Figure 2B, right panel) that was done with a Fluorolog Horiba spectrofluorometer. Anisotropy was recorded at the maxima for emission of Pa-aIF1* at 473 nm (λ_exc_ = 428 nm). For each titration point, 10 anisotropy measurements were recorded successively at different times in order to verify that equilibrium has been reached. Aliquots (1.7 μl) of 30S or 30S:mRNA solutions were added for titration. 30S:mRNA complex was prepared by mixing 30S with a 2-fold molar excess of mRNA. After saturation, a reverse titration was performed with unlabeled protein to verify that the labeled and unlabeled proteins compete for a same binding site (Supplementary Figure S2).

Finally, fluorescence anisotropy (*r*) was plotted as a function of 30S or 30S:mRNA concentration and fitted as described in (42) by non-linear regression (Origin, OriginLab Corp., Northampton, USA) using a quadratic equation for the binding of Pa-aIF1 to the 30S subunit (42):

\begin{eqnarray*}r\ &=& {r_{aIF1*}}\ \ + \ \left[ {{r_{30S:aIF1*}} - {r_{aIF1*}}} \right]\nonumber\\ && \, \frac{{\left( {\left[ {aIF1*\left] + \right[30S} \right] + Kd} \right) - \sqrt {{{\left( {\left[ {aIF1*\left] + \right[30S} \right] + Kd} \right)}^2} - \ 4\left[ {aIF1*} \right]\left[ {30S} \right]} }}{{2\left[ {aIF1*} \right]}}\end{eqnarray*}

where [aIF1*] is the total concentration of initiation factor, [30S] is the concentration of small ribosomal subunits (or of 30S:mRNA complex), *K*_d_ is the dissociation constant of Pa-aIF1* from its complex with 30S (or with 30S:mRNA), r_aIF1*_ is the fluorescence anisotropy of Pa-aIF1* and r_30S:aIF1*_ is the fluorescence anisotropy of the 30S:aIF1* complex. For all titrations, it was verified that fluorescence emission did not change upon binding and therefore no correction for changes in quantum yield have been performed.

A similar protocol has been used to measure binding affinities of a 3′-fluorescein labeled mRNA (wt-aEF1A-mRNA-3′Fl, Dharmacon) to 30S and 30:aIF1 complex. This model mRNA corresponds to the 3′ region of the mRNA encoding Pa-aEF1A with the sequence _(-17)_AUUU**GGAGGUGAU**UUAA**AUG**CCAAAG_(+9)_. Fluorescence anisotropy was followed at 530 nm (λexc = 485) during titration of mRNA-3′Fl with successive additions of purified Pa-30S or Pa-30S:aIF1 complex. Pa-30S:aIF1 complex was prepared by mixing 30S at a concentration of 1 μM with a two-fold molar excess of Pa-aIF1. Concentrations used are indicated in the legend of Figure 2. Anisotropy was plotted as a function of 30S or 30S:aIF1 complex and fitted by a quadratic equation as described above. After saturation, a reverse titration was performed with unlabeled mRNA to verify that the labeled and the unlabeled mRNA compete for a same binding site.

### Toeprinting analyses of translation initiation complexes

A DNA fragment containing the Pa-aEF1A ORF and 36 upstream nucleotides was cloned between the XbaI and XhoI sites of a pET3a derivative. The plasmid was linearized with NcoI, transcribed using T7 RNA polymerase, as described (43) and purified on a mono-Q column (GE Healthcare).

Toeprinting experiments were performed essentially as described (7,8,43,44). 30S complexes were assembled on aEF1A mRNA (or their variants) and were analyzed by primer extension using AMV reverse transcriptase (Invitrogen). A typical mixture for 20 reactions was made as follows. Six picomoles of 5′-labeled (Dye-682 fluorophore, Eurofins) primer were annealed to 2.4 pmol of mRNA in 40μl of annealing buffer (60 mM NH_4_Cl, 10 mM HEPES pH 7.5, 7 mM 2-mercaptoethanol), in the presence of 40 units of RNase inhibitor (Invitrogen). After heating 3 min at 60°C, the reaction was cooled at room temperature and Mg(CH_3_COO)_2_ was added to reach a concentration of 60 mM. A premix (90 μl) was prepared by adding to 40 μl of the above mixture, 20 μl of 30S subunits at a concentration of 240 nM, 10μl of dNTPs (3.75 mM each), 10 μl GDPNP 5 mM and 120 units RNase inhibitor. After a 5 min incubation at 51°C, the premix was distributed as 4.5 μl aliquots into 4 μl mixtures containing factors and Met-tRNA as desired (factors were prediluted to 2.5 μM in buffer B and Met-tRNA was prediluted to 10 μM in water). Each tube was incubated 10 min at 51°C, before addition of 1 μl of AMV Reverse Transcriptase (2 units/μl, Invitrogen) and further incubated 15 min at 51°C. The reactions were quenched by adding 3 μl of stop solution containing 95% formamide and blue dextran. A typical reaction mixture (9.5 μl) contained 0.12 pmol of mRNA (12.6 nM), 0.24 pmol of 30S (25.2 nM), 2.77 pmol of initiation factors (263 nM), 10.5 pmol of Met-tRNA (1052 nM) in a buffer containing 10 mM HEPES pH 7.5, 158 mM NaCl, 20 mM NH_4_Cl, 13.5 mM MgAcetate, 0.05 mM Spermine, 0.26 mM GDPNP and 0.2 mM dNTP. Reverse transcripts were analyzed on a Licor 4200 DNA sequencer. Sequencing reactions made with the corresponding DNA plasmid and the RT primer were loaded in order to identify the toeprinting positions. Finally, the images were processed using the ImageJ software for quantitative analysis (45).

## RESULTS

### 3D structure of *Methanocaldococcus jannaschii aIF1*

**Figure 1.**
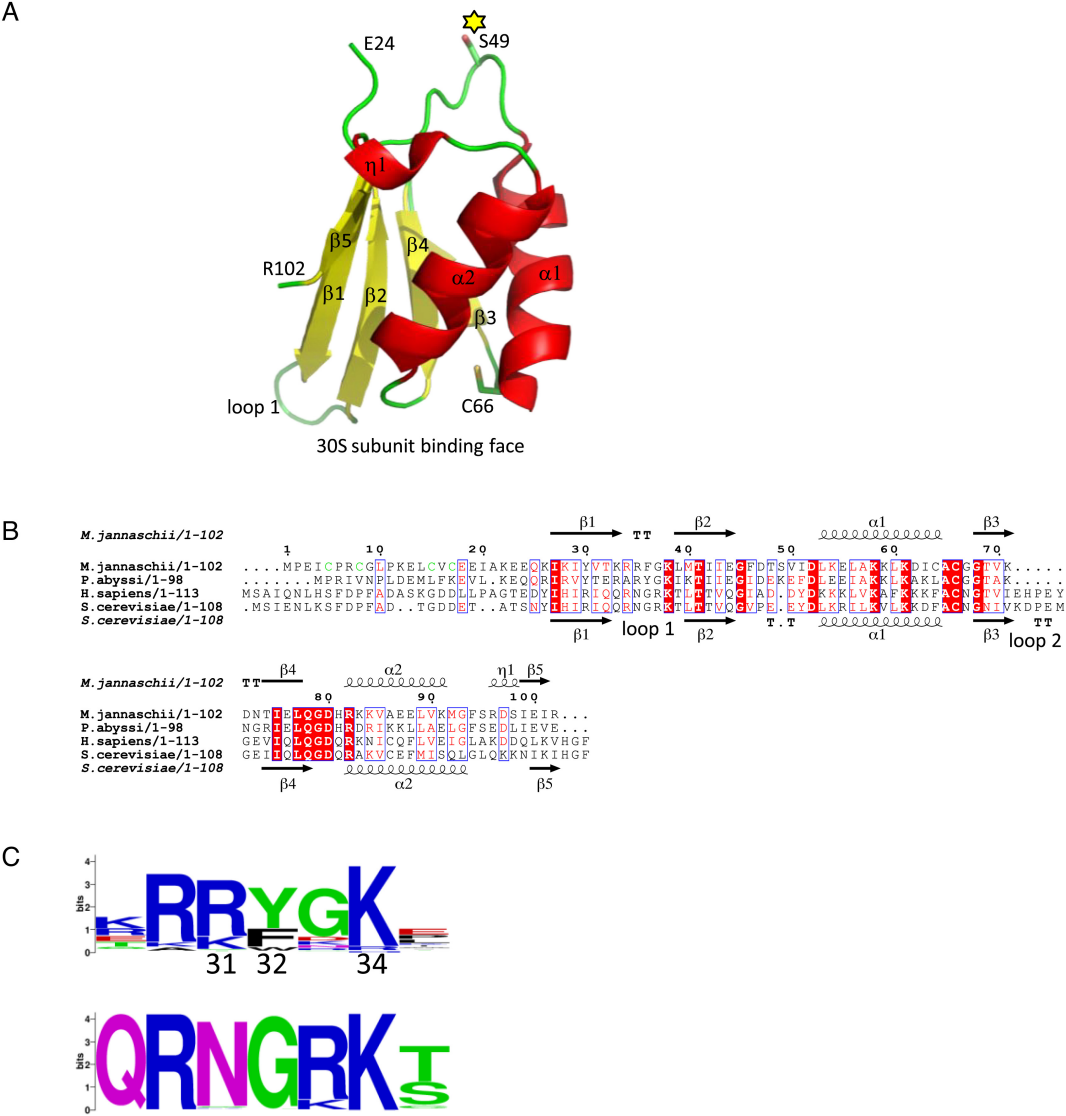
Structure of archaeal aIF1. (**A**) Crystal structure of aIF1 from *Methanocaldococcus jannaschii*. Secondary structures are colored: helices in red, β strands in yellow and loops in green (N and C residues are indicated). S49 and the conserved C66 are in stick. S49 corresponds to K45 in Pa-aIF1. This position has been mutated into C and labeled with coumarin in Pa-aIF1*. (**B**) Structural alignment of some archaeal (*M. jannaschii* and *P. abyssi*) and eukaryotic (*H. sapiens* and S*. cerevisiae*) a/eIF1. Secondary structures refer to Mj-aIF1 crystallographic 3D-structure (top) or to the NMR *S. cerevisiae* eIF1 structure (PDB ID code 2OGH). The cysteine residues forming a putative zinc finger in the N-domain of Mj-aIF1 are colored in green. Panels A and B were drawn using Pymol (http://www.pymol.org) and Espript, respectively (64). (**C**) Sequence logos (https://weblogo.berkeley.edu/logo.cgi) of loop 1 in archaea (upper logo, calculated from 744 sequences) and in eukaryotes (lower logo, calculated from 1118 sequences). Numbering is from *P. abyssi* aIF1.

The crystallographic structure of *Methanocaldococcus jannaschii aIF1* (Mj-aIF1) was solved by SIRAS and refined to 2.1 Å resolution (Material and Methods and Supplementary Table S1). Residues 24–102 of Mj-aIF1 form a five-stranded antiparallel β-sheet with two α-helices and one short 3^10^ helix on its convex face (Figure 1A). The overall fold of the archaeal factor is highly similar to those of yeast and human eIF1 (rmsd = 2.73 Å for 66 Cα atoms compared or 1.46 Å for 59 Cα atoms compared). It is also highly similar to the recently published aIF1 structure from *Pyrococcus horikoshii* (46), rmsd = 0.62 Å for 61 Cα atoms compared). The N-terminal part of aIF1 (residues 1 to 23) is not visible in the electron density suggesting that, as for the eukaryotic eIF1 counterparts (47,48), the N-terminal part of the factor is mobile with respect to the rest of the protein outside of the ribosomal initiation complex. According to the similarities of the folds, sequence alignments of eukaryotic and archaeal a/eIF1 show high level of identities between representatives of both kingdoms. The most variable parts when comparing eukaryotic and archaeal factors are located in the N- and C-terminal extremities as well as within the β1–β2 (loop 1) and β3–β4 (loop 2, specific to eukaryotic eIF1) connecting loops (Figure 1B and C). Notably, 64% of 744 aligned archaeal aIF1 sequences, comprising all those from Methanococcales (including that of Mj-aIF1), have a sequence motif in the N-domain containing four cysteine residues and forming a putative zinc knuckle of the form CX_2_CGLPX_3_CϕC (where ϕ stands for V, I, M or A; Figure 1B and Supplementary Table S2). Indeed, after dialysis against a buffer containing 0.1 mM EDTA, Mj-aIF1 retained 0.8 ± 0.05 μM Zn per μM of protein, as measured using atomic absorption spectroscopy (Material and Methods and Supplementary Figure S1C). This value agrees with one Zn site per polypeptide, taking into account the uncertainty in protein concentration determination arising from the low computed extinction coefficient of Mj-aIF1 that do not contain any Trp residue. This strongly argues in favor of a zinc knuckle in the N-domain of these aIF1s. As a control, we verified that no zinc was detectable in Pa-aIF1.

### Bindings of Pa-aIF1 and mRNA to the small ribosomal subunit are synergistically coupled

**Figure 2.**
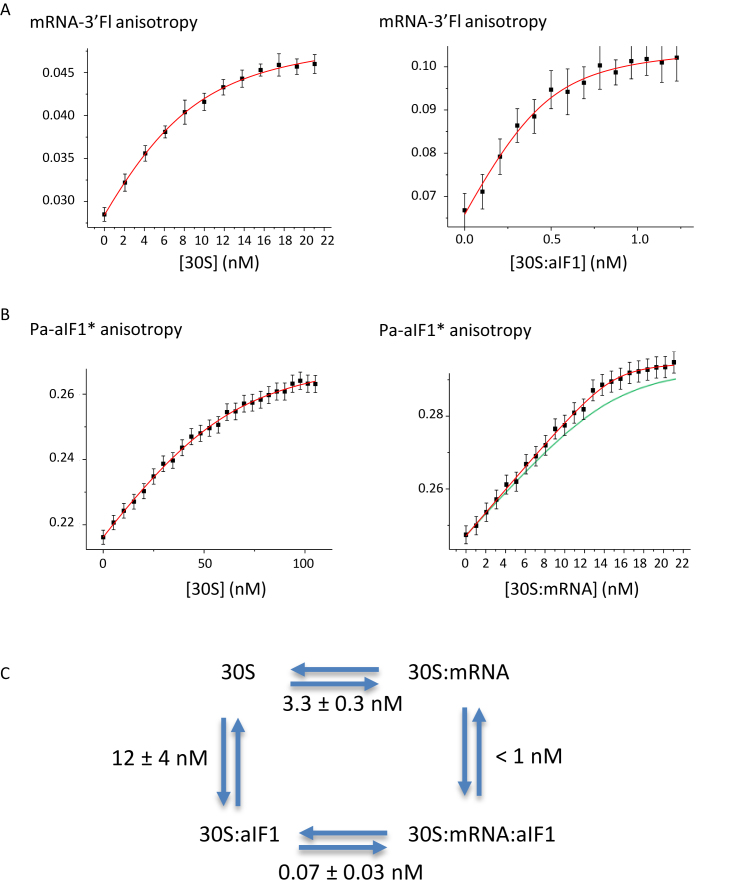
Thermodynamic framework for the binding of Pa-aIF1 and mRNA to Pa-30S. (**A**) Binding of mRNA-3′Fl to Pa-30S and to Pa-30S:aIF1 complexes. Fluorescence anisotropy of mRNA-3′Fl was followed during titration with purified Pa-30S. Left, concentration of Pa-30S was varied from 2 to 22 nM and concentration of mRNA-3′Fl was 8 nM. Right, concentration of Pa-30S:aIF1 was varied from 0.1 nM to 1.22 nM and concentration of mRNA-3′Fl was 0.5 nM. (**B**) Binding of Pa-aIF1* to Pa-30S and to Pa-30S:mRNA complexes. Fluorescence anisotropy of Pa-aIF1* was followed during titration with purified Pa-30S. Left, concentration of Pa-30S was varied from 5.15 nM to 105 nM and concentration of Pa-aIF1* was 50 nM. Right, concentration of Pa-30S:mRNA was varied from 1 to 23.7 nM and concentration of Pa-aIF1* was 13 nM; the red curve represents the best fit of the data with a *K*_d_ of 0.2 nM whereas the green one represents a theoretical curve with a *K*_d_ of 1 nM. This shows that the *K*_d_ value is lower than 1 nM. For A and B, results were fitted using standard equations for *K*_d_ determination (Material and Methods). (**C**) Thermodynamic framework for the binding of Pa-aIF1 and mRNA to Pa-30S showing that the bindings of mRNA and Pa-aIF1 to the 30S are synergistically coupled.

In a first series of experiments, we measured the binding of a model mRNA to purified Pa-30S at 51°C using fluorescence anisotropy. We used a synthetic 26-nucleotide-long mRNA labeled at its 3′ extremity with fluorescein. The sequence corresponds to the natural start region of the mRNA encoding the elongation factor aEF1A from *P. abyssi*, _(–17)_AUUU**GGAGGUGAU**UUAA_-1_**A_1_UG**CCAAAG_(+9)_, which contains a strong Shine-Dalgarno sequence (wt-aEF1A-mRNA-3′Fl, Dharmacon). Fluorescence anisotropy was followed during titration of mRNA-3′Fl with successive additions of purified Pa-30S (Figure 2A, left panel). A Kd value of 3.3 ± 0.3 nM was deduced (Material and Methods). As a control, a reverse titration of the Pa-30S:Fl-mRNA complex with unlabeled mRNA caused, as expected, a decrease in anisotropy showing that the unlabeled mRNA and mRNA-3′Fl compete for the same binding site in the 30S (data not shown). We then followed the binding of mRNA-3′Fl to a preformed 30S:aIF1 complex (Figure 2A, right panel) and derived a *K*_d_ value of 0.07 ± 0.03 nM. Overall, the data indicate that aIF1 favors the binding of the mRNA to the 30S by a factor of nearly 50.

We then labeled Pa-aIF1 to measure its binding affinity to the 30S and to the 30S:mRNA complex. Native Pa-aIF1 contains a single cysteine residue (C62). However, because C62 is located in the 30S:aIF1 binding site (24), its labeling with a bulky group was likely to impair binding of the protein to the small ribosomal subunit. Consistent with this prediction, no anisotropy variation was observed when a wt-Pa-aIF1 coumarin derivative was titrated by the Pa-30S:mRNA complex. Therefore, we introduced a cysteine residue in a variable loop of Pa-aIF1 opposite to the 30S binding site (Figure 1A) and changed C62 into S. Notably, S or T residues are encountered at this position in 21% of 753 aligned aIF1 sequences. The K45C-C62S variant was produced and the corresponding protein was labeled with coumarin (Material and Methods). The labeled protein is hereafter named Pa-aIF1*. A *K*_d_ value of 12 ± 4 nM was deduced by titrating Pa-aIF1* (50 nM) with the 30S subunit (Figure 2B). With the same Pa-aIF1* concentration but in the presence of mRNA, stoichiometric binding of the factor to the 30S:mRNA complex occurred along the titration, showing a much higher binding affinity. Unfortunately, because of the sensitivity of the assay, the concentration of Pa-aIF1* could only be decreased to 13 nM in the titration. From this experiment, a *K*_d_ value lower than 1 nM was deduced (Figure 2). This showed that the binding of aIF1 to the 30S is favored by at least one order of magnitude when the ribosomal subunit is already bound to the mRNA. Finally, as a control, reverse titrations of the Pa-30S:mRNA:aIF1* and Pa-30S:aIF1* complexes with unlabeled factor showed that the coumarin label had no significant influence on the binding affinity of aIF1 (Supplementary Figure S2). Overall, in agreement with the reciprocal effect described above, the thermodynamic framework (Figure 2C) indicates that the binding of aIF1 and that of the mRNA to the 30S are synergistically coupled. This coupling can either arise from a direct interaction between both partners or be indirectly induced by conformational changes in the 30S upon binding of one partner.

### Binding of mRNA to the 30S:aIF1 complex depends on the Shine-Dalgarno sequence

**Figure 3.**
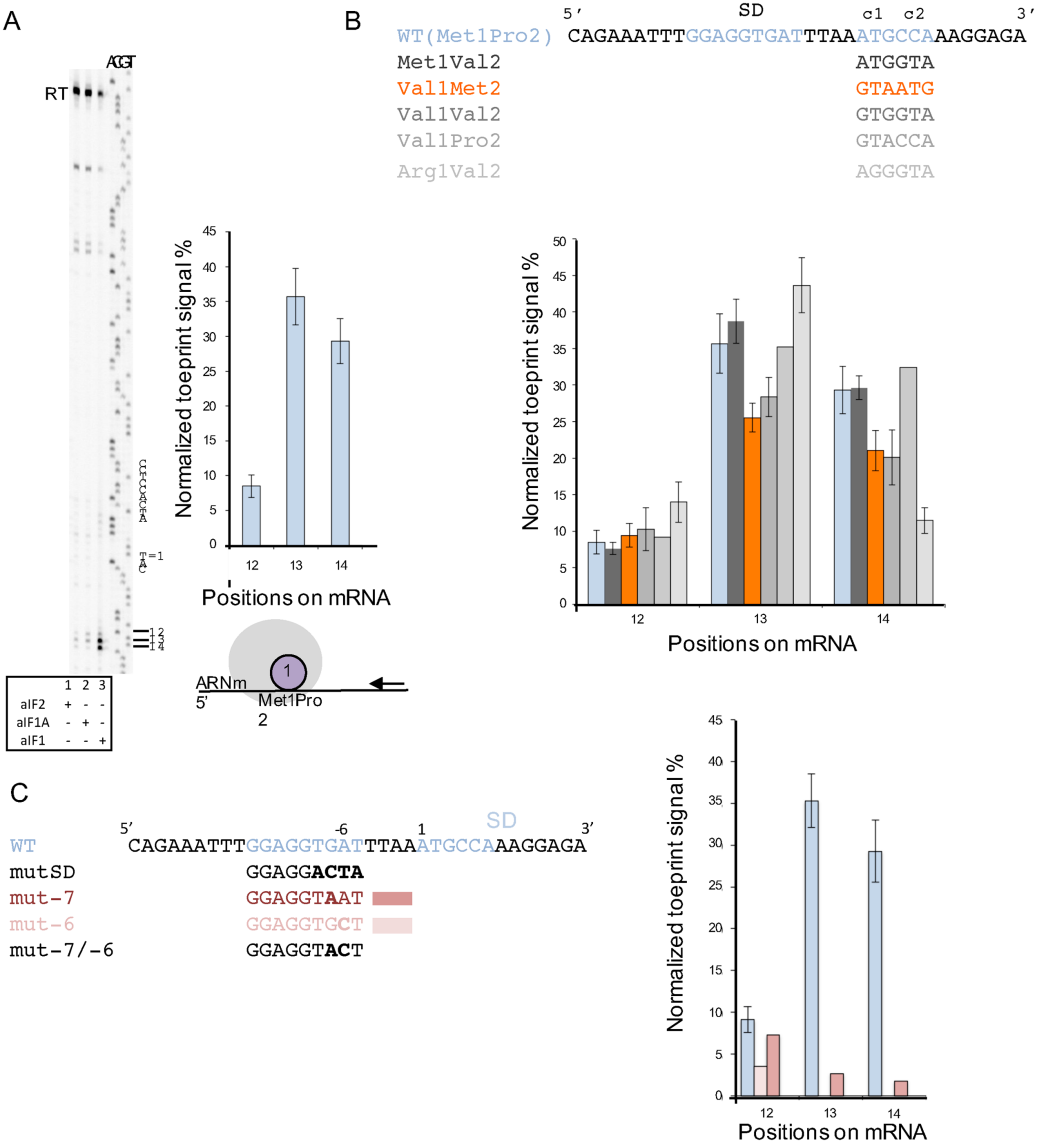
Activity of Pa-aIF1 in the stabilization of 30S:mRNA complexes. (**A**) Toeprinting analysis of ribosomal complexes assembled on aEF1A mRNA in the presence of Pa-30S and of the indicated initiation factor. The positions of the initiation codon and of the Shine-Dalgarno sequence of aEF1A-mRNA are shown to the right of the reference lanes, which correspond to the aEF1 sequence derived using the same primer. Full-length cDNA is marked RT. The normalized toeprint signal for 30S:mRNA:aIF1 complex on positions +12, +13, +14 obtained after data processing using ImageJ is shown to the right of the view. The means of 13 independent experiments with the calculated standard deviations are represented. (**B**) Normalized toeprint signals for 30S:mRNA:aIF1 complex on positions +12, +13, +14 obtained with various mRNAs in which the sequences of the first and second codons have been varied. (**C**) Normalized toeprint signals for 30S:mRNA:aIF1 complexes on positions +12, +13, +14, obtained with various mRNAs in which the SD sequence has been varied. In B and C; means and standard deviations are calculated from at least two independent experiments, except for Val1-Pro2, mut -7 and mut -6 where values are derived from a single experiment. No significant toeprint signals were obtained with mut SD and mut-7/-6 (Supplementary Figure S4).

To better understand how the mRNA influences aIF1 binding to the 30S subunit, toeprinting experiments were performed. We used a 600-base-long *in vitro* transcribed RNA fragment corresponding to the elongation factor Pa-aEF1A mRNA. This mRNA contains the 26-nucleotide-long sequence used in fluorescence anisotropy measurements. First, the influence of each initiation factor on the toeprinting signal was investigated. Only faint bands could be detected when aIF1A or aIF2 were added. However, a clear signal was observed with the 30S:mRNA:aIF1 complex (Figure 3A). The toeprinting signal mainly appeared at positions +12, +13, +14, 3′ to the AUG codon (A = 1). We verified that increasing concentrations of Pa-aIF1 in the toeprinting experiment did not change the RT arrest signal showing that toeprint experiments were performed with a saturating concentration of Pa-aIF1 with respect to Pa-30S (Supplementary Figure S3). In order to investigate the influence of the AUG codon on the toeprinting signal, we designed mutated mRNAs carrying various codon sequences in place of the wild-type met1pro2 sequence. As shown in Figure 3B, whatever the mRNA sequence, the toeprinting signal due to the 30S:aIF1 complex is clearly visible on the three major positions +12, +13, +14 as observed with the wt-aEF1A-mRNA. Some slight variations are however observed as compared to the WT-aEF1A-mRNA signal. Overall, 30S:aIF1 toeprinting signal is essentially independent on the position of the methionine codon or on the presence of a met codon.

Our model mRNA contained a Shine-Dalgarno sequence (_-13_GGAGGUGAU_-5_) complementary to nine bases of the *P. abyssi* 16S rRNA 3′-end. The corresponding nine base pairs were indeed visible in the archaeal PIC cryo-EM structures (24). The GGAGG motif makes part of a consensus sequence motif of the RBSs that was extracted from the upstream regions of 508 coding sequences of the *P. abyssi* transcriptome (49). In order to test the contribution of the four bases following the GGAGG sequence to the stability of the ribosomal 30S:mRNA:aIF1 complex, we first mutated the wild-type mRNA aEF1A sequence in these four bases and purified the corresponding mRNA (mutSD, Figure 3C). Using this mutated mRNA, no toeprint signal was observed in the presence of aIF1. We then used another mRNA corresponding to the natural 5′ region of Pa-aIF2γ containing an SD consensus sequence differing by bases –5 and –8 from that of wt-aEF1A-mRNA (_-13_GGAGG**C**GA**G**_-5_). Using Pa-aIF2γ mRNA, a toeprint signal of similar amplitude to that obtained with wt-eEF1A-mRNA was observed though at positions +13 to +15 (data not shown). We finally mutated either base –6 or base –7 or both in wt-eEF1A-mRNA and observed that both bases indeed contribute to the stability of the 30S:mRNA:aIF1 complex (Figure 3C and S4). This was confirmed by fluorescence anisotropy measurements showing a value of 100 ± 25 nM for the dissociation constant of the mutant mRNA-6/-7 from its complex with 30S:aIF1 as compared to <1 nM for the wt-mRNA (Supplementary Figure S4). Overall, the data shows that Pa-aIF1 dependent mRNA binding is strongly sensitive to the SD sequence. It is notable that the –5 to –7 nucleotidic sequence is not much conserved in *P. abyssi* (49) or in *S. solfataricus* (50). Our results strongly suggest that additional base pairing between the –5 to –8 sequence of the mRNA with the 16S rRNA contributes to the high level of expression of the gene coding for EF1A.

### Pa-aIF1 favors a dynamic initiation complex in the presence of the Pa-aIF2 ternary complex

In a second step, we studied the effect of aIF1 on the stability of the full preinitiation complex, 30S:mRNA:aIF1:aIF1A:aIF2:GDPNP:met-tRNA (PIC). Previous studies have shown that *E. coli* initiator Met-tRNA_f_^Met^A_1_-U_72_ variant was a good mimic of Pa-Met-tRNA_i_^Met^ with identical acceptor stem and anticodon loop (Supplementary Figure S5, (24,30,40)). Therefore, because Met-tRNA_f_^Met^A_1_-U_72_ tRNA can be obtained with a much higher purity than Pa-Met-tRNA_i_^Met^ (40), toeprinting experiments were performed with *E. coli* initiator Met-tRNA_f_^Met^A_1_-U_72_. Control experiments showed very similar toeprint signals with Met-tRNA_f_^Met^A_1_-U_72_ as compared to Pa-Met-tRNA_i_^Met^ (Supplementary Figure S5B).

**Figure 4.**
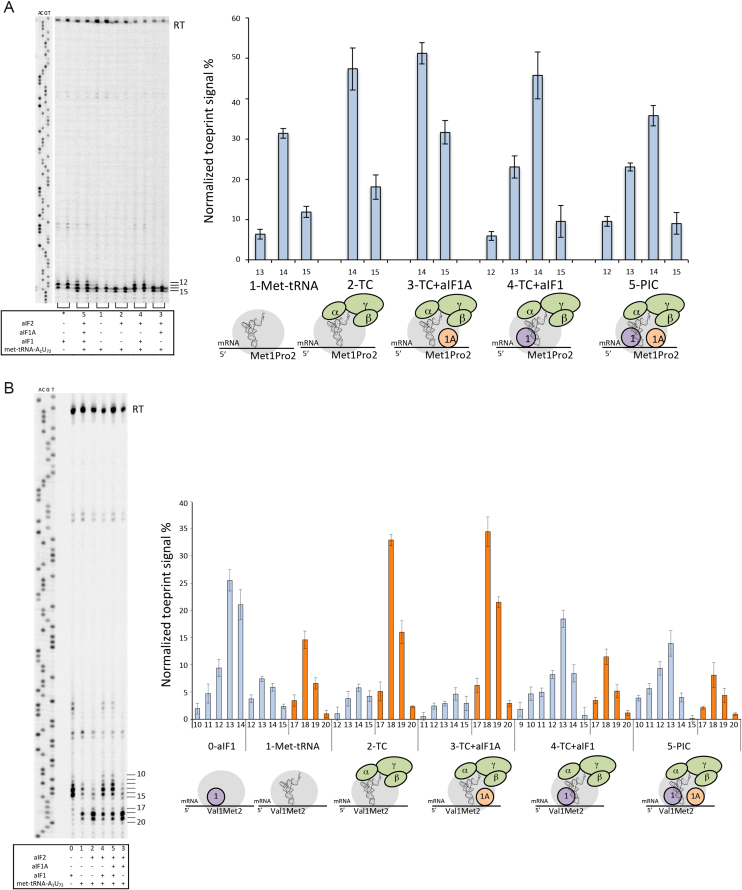
Toeprinting analysis of 30S initiation complexes. (**A**) Toeprinting analysis of 30S initiation complexes on wt-aEF1A-mRNA. Left: a typical experiment is shown. Toeprinting positions are indicated to the right of the gel. Right: normalized toeprint signals obtained with 30S subunits plus various combinations of initiation factors and tRNA, as indicated below the graph. (**B**) Toeprinting analysis of 30S initiation complexes assembled on Val1Met2-aEF1A-mRNA. Left: a typical experiment is shown. Toeprinting positions are indicated to the right. Right: Analysis of toeprinting signals obtained with various combinations of initiation factors and tRNAs. PIC = full pre-initiation complex. TC = ternary complex aIF2:GDPNP:Met-tRNA_f_^Met^A_1_-U_72_. TC+aIF1A and TC+aIF1 correspond to PIC-w/o-aIF1 and PIC-w/o-aIF1A, respectively. Means and standard deviations are calculated from at least 3 independent experiments.

A complex consisting of the 30S ribosomal subunit, the model wt-aEF1A-mRNA and Met-tRNA_f_^Met^ A_1_-U_72_ caused toeprint signals at positions +13 to +15 with positions +14 and +15 being the strongest (Figure 4A, lane 1). Notably, under displacement of the AUG codon to position 2, as in mRNA Val1Met2, the main signal shifted to positions +18, +19 (Figure 4B, lane 1). Therefore, with wt-aEF1A-mRNA, toeprint signals at positions +14 and +15 can be ascribed as reflecting a complex with the initiator tRNA base-paired with the AUG codon in the P site. Similarly, positions +18 and +19 reflects a complex with the initiator tRNA base-paired with the AUG codon in the P site using the Val1Met2-aEF1A mRNA.

In the presence of the ternary complex, aIF2:GDPNP:Met-tRNA_f_^Met^A_1_-U_72_, the toeprint signal increased, as compared to that obtained with the tRNA alone, and became restricted to positions +14 and +15 (Figure 4A, lane 2). The effect of aIF2 required methionylation of the initiator tRNA, consistent with the fact that affinity of aIF2 for tRNA strongly depends on its aminoacylation with methionine (30). Indeed, addition of aIF2 led to an increase by 60% of the signal at +15 +16 in the case of Met-tRNA whereas it had no detectable effect with non-aminoacylated tRNA (data not shown). In the presence of aIF1A and of the TC, a moderate but reproducible increase of the RT arrest signal at position +15 was observed (Figure 4A, lane 3). This showed that aIF1A helped to stabilize the initiator tRNA in the P site when aIF1 was not present (Figure 4A, compare lanes 2 and 3). In contrast, addition of aIF1 to the 30S:mRNA:TC complex strongly decreased the toeprint signal at position +15 while signal appeared at positions +12 and +13 (Figure 4A, lane 4). In the presence of all initiation factors (full preinitiation complex, PIC; Figure 4A, lane 5) the signal at positions +14 and +15 was further lowered indicating that the conformation of the complex became even more dynamic when both aIF1 and aIF1A were present (Figure 4A, compare lanes 4 and 5).

The same experiments were performed in the context of Val1Met2-aEF1A-mRNA. Similar results were obtained (Figure 4B). Briefly, in the presence of the TC only, the main signal was observed at positions +18 and +19, showing the adjustment of the ribosomal complex on the met2 codon. Addition of aIF1A further reinforced the signal at position +19 (Figure 4B, compare lanes 2 and 3). In contrast, addition of aIF1 to the TC decreased the toeprinting intensity at positions +18, +19 (Figure 4B, lane 4). As for wt-mRNA, this effect was reinforced in PIC, when both aIF1 and aIF1A were present (Figure 4B, compare lanes 4 and 5).

Overall, the data indicate that in the absence of aIF1, the initiation complex is stably bound to the mRNA with met-tRNA base-paired to the AUG start codon (Figure 4, lanes 3). When aIF1 is present, the initiation complex has a more dynamic conformation reflecting that the met-tRNA is not stably base-paired to the start codon (Figure 4, lanes 4 and 5). Therefore, the results presented in this section show that aIF1 impairs stable tRNA accommodation within the P site. Notably, in the presence of aIF1, aIF1A reinforces the dynamic character of the PIC whereas it favors stable binding of the tRNA in the P site in the absence of aIF1. This shows that the two factors act cooperatively during start codon selection.

### Residues of the basic loop are involved in the binding of aIF1 to the 30S

Previous structural studies of eukaryotic or archaeal translation initiation complexes have identified the binding site of e/aIF1 on the small ribosomal subunit (14,15,17,18,24). The basic loop, also called loop 1, located between strands β1 and β2 was proposed to carry residues in contact with the 18S or 16S rRNA (15,17,24,51). Three Pa-aIF1* variants with mutated basic residues in loop 1 were produced. Binding affinities of these variants for the 30S and the 30S:mRNA complex were measured by fluorescence anisotropy (Table 1). All mutations affected the binding affinity of aIF1* for the 30S:mRNA complex. Mutations R31A and K34A affected the binding affinity of aIF1* to both 30S and 30S:mRNA but the mRNA still favored their binding to the 30S by factors of 36 and at least of 6.6, respectively (Table 1). This argues in favor of an interaction of R31 and K34 with the 30S. Notably, the binding affinity of aIF1*-Y32A was similar for the 30S and the 30S:mRNA complex, showing that the synergistic binding of aIF1 and of the mRNA to the 30S had been lost. Overall, some residues of the basic loop, such as R31 and K34 are involved in the binding to the 30S whereas others, as Y32, are necessary to stabilize the 30S:mRNA conformation.

**Table 1.** Binding of Pa-aIF1* and mutants to the 30S and 30S: mRNA. Fluorescence anisotropy of Pa-aIF1* and its variants was followed during titration with purified Pa-30S or Pa-30S:mRNA complexes as described in Material and Methods and in the legend of Figure 2. Concentrations of Pa-aIF1* variants used in the titrations were 50 nM. For aIF1*-R31A, concentration of Pa-30S was varied from 20 to 266 nM and concentration of Pa-30S:mRNA was varied from 5.15 nM to 108.8 nM. For aIF1*-Y32A, concentrations of Pa-30S and Pa-30S:mRNA were from 5.15 nM to 108.8 nM. For aIF1*-K34A, concentrations of Pa-30S and Pa-30S:mRNA were from 20 to 266 nM

Kd (nM)	30S	30S:mRNA
Pa-aIF1*	12 ± 4	<1
Pa-aIF1*-R31A	84 ± 6	<3
Pa-aIF1*-Y32A	20 ± 5	22 ± 4
Pa-aIF1*-K34A	>300	45 ± 9

In a second step, we used variants of wt-Pa-aIF1 at positions Y32 and K34 in toeprinting experiments. As shown in Supplementary Figure S3, the signal pattern obtained with Pa-aIF1-K34A resembled that obtained with wt-Pa-aIF1 but an 8-fold higher factor concentration was necessary, in agreement with the reduced binding affinities of this variant (Table 1). This shows that Pa-aIF1-K34A is still able to stabilize the 30S:mRNA complex. Interestingly, toeprinting signals obtained with Pa-aIF1-Y32A were very weak and did not markedly vary with the concentration of the factor. This observation is consistent with *K*_d_ values and gives further credit to the idea that of Y32 participates in the stabilization of the 30S:mRNA conformation.

### Pa-aIF1 favors the rejection of an elongator tRNA by the initiation complex

**Figure 5.**
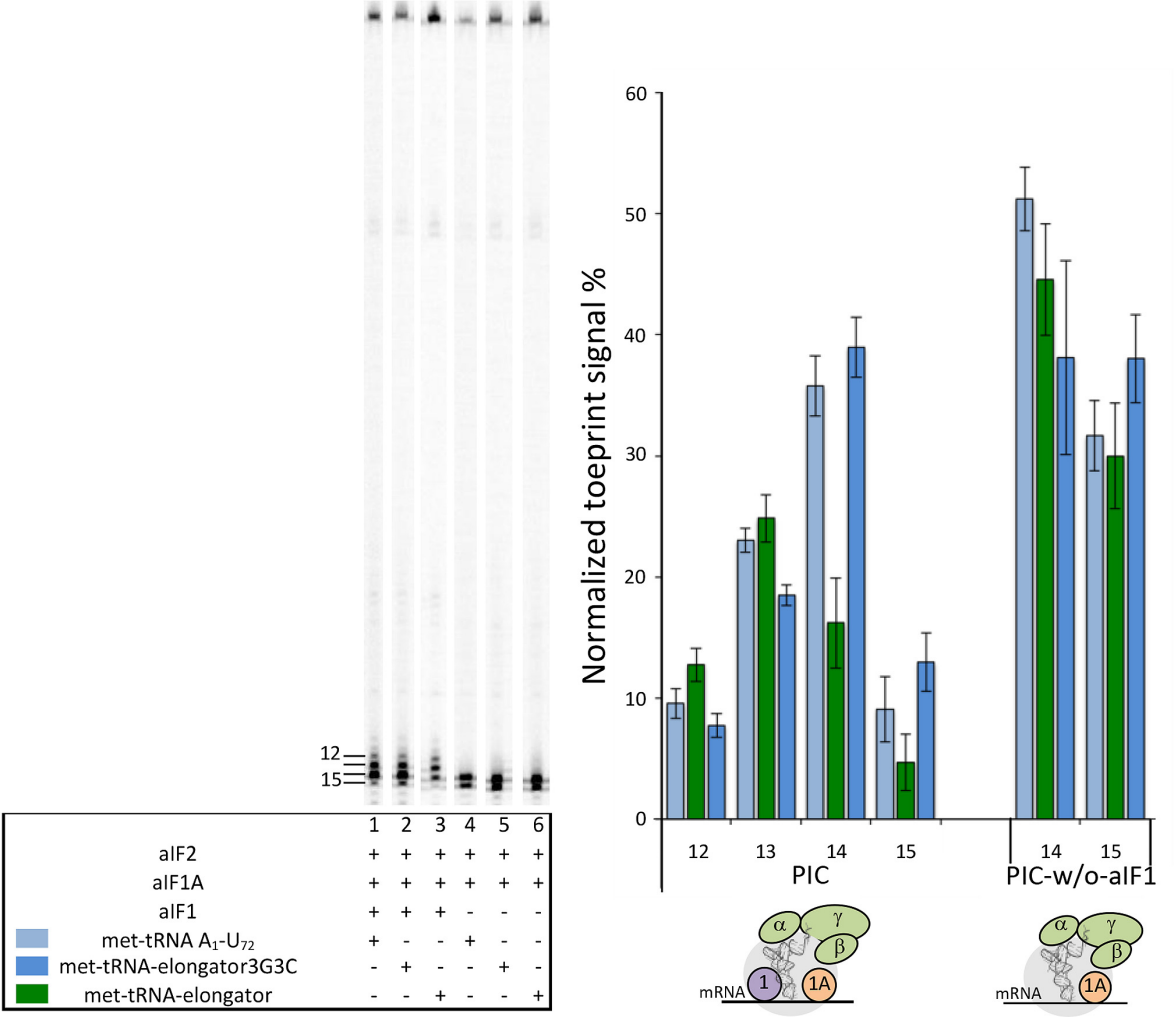
aIF1 discriminates a PIC assembled with an elongator-Met-tRNA from a PIC assembled with a Met-tRNA carrying three GC base pairs in the anticodon stem. Toeprinting analysis of PIC and PIC- w/o-aIF1 complexes assembled on wt-aEF1A mRNA in the presence of Met-tRNA A_1_-U_72_ (light blue), Met-tRNA elongator (green), Met-tRNA elongator 3G3C (dark blue). The toeprinting positions are indicated below the graph and to the left of the gel. Means and standard deviations are calculated from at least 3 independent experiments.

The ability of aIF1 to impair stable tRNA accommodation within the P site is likely to be favorable to the edition of wrong initiation complexes. Such complexes are characterized by a tRNA weakly bound in the P site. This can occur because of the absence of codon:anticodon base pairing or because the tRNA is an an elongator tRNA devoid of the 3 GC base pairs in the anticodon stem, known to improve the P site tRNA affinity (52,53). In order to probe the capacity of Pa-aIF1 to impair stabilization of wrong initiation complexes, we took advantage of the previous observation that Pa-aIF2 significantly binds elongator Met-tRNA^Met^, with a *K*_d_ of 450 nM as compared to 90 nM Met-tRNA_f_^Met^A_1_-U_72_ (30). This allowed us to use a ternary complex made with elongator Met-tRNA^Met^ in toeprinting experiments and to test the effect of aIF1 on the accommodation of the erroneous tRNA. Notably, in the absence of aIF1 (PIC-w/o-aIF1), an initiation complex with the elongator Met-tRNA_m_^Met^ showed similar toeprinting signals at position +14 and +15 as the complex made with initiator Met-tRNA_f_^Met^A_1_-U_72_ (Figure 5, lanes 4 and 6). Upon addition of aIF1 however, the intensity of the toeprinting signal at positions +14, +15 was much lower for the PIC complex with the elongator Met-tRNA than that observed for the PIC complex with the initiator Met-tRNA_f_^Met^A_1_-U_72_. This illustrates that aIF1 indeed disfavors accommodation of an erroneous initiation complex made with an elongator tRNA.

The elongator Met-tRNA lacks the three GC base pairs of the anticodon stem characteristic of initiator tRNAs (Supplementary Figure S5). Many studies have shown that the 3 GC base pairs were important for the selectivity of the initiator tRNA during translation initiation. Therefore, the different effects of aIF1 on PIC made with either initiator or elongator tRNA can be linked to the absence of the three GC base pairs. In order to test this idea, we used a mutant elongator Met-tRNA possessing the three consecutive GC base pairs in the anticodon stem (Supplementary Figure S5). Using this tRNA (met-elongator-tRNA3G3C, Figure 5), we observed that the toeprint signals were similar to those obtained with the met-initiator tRNA in the PIC. This demonstrates that the selectivity of the pre-initiation complex for the initiator tRNA is linked to the 3GC base pairs of the anticodon stem of the tRNA.

## DISCUSSION

Archaeal translation initiation uses three initiation factors, aIF1, aIF1A and aIF2, homologous to their eukaryotic counterparts. This led to the definition of a structural core, common to eukaryotes and archaea that controls translation initiation accuracy. This core is made up of the small ribosomal subunit, the mRNA, the Met-tRNA_i_^Met^ and the three factors e/aIF1, e/aIF1A and e/aIF2. Recently stable archaeal PIC intermediates were analyzed by cryo-EM (24). These studies have in particular shown direct interactions between the initiation factors within the PIC and have highlighted a direct role of aIF2 in the selection of the start codon. In the present study, we used *in vitro* reconstituted ribosomal complexes in the *P. abyssi* system to gain insight in the role of aIF1 in the archaeal translation initiation process.

Using fluorescence anisotropy, we measured the affinities of Pa-aIF1 and of the mRNA for the *P. abyssi* 30S ribosomal subunit and showed that the binding of the two partners is cooperative. Therefore, aIF1 helps to stabilize the conformation of the 30S:mRNA complex. This is consistent with the position of the factor observed in the cryo-EM structures (Supplementary Figure S6, 24). Then, using toeprinting experiments, we observed a strong AMV RT arrest signal due to formation of the 30S:mRNA:aIF1 complex. Variations of the sequence of the mRNA further indicated that arrest signals with 30S:mRNA:aIF1 complexes mainly depended on the strength of the Shine-Dalgarno sequence and were at most marginally influenced by the presence or the position of the AUG codon (Figure 3). The stabilizing effect of Pa-aIF1 on the 30S:mRNA complex observed in the present study is in agreement with previous results obtained with the *S. solfataricus* system suggesting that Ss-aIF1 stimulated binding of a model RNA to the ribosome and increased translation level (25). Interestingly, our conclusions are reminiscent of recent studies on eukaryotic systems showing that translation efficiency was linked to eIF1 availability. More precisely, low levels of eIF1 were associated to translation initiation at start sites embedded in a suboptimal nucleotidic context (54). In the same view, the autoregulative negative feedback loop in which a high level of eIF1 inhibits its own translation is caused by an AUG codon in a poor context (55,56). Therefore, it appears that in both archaea and eukaryotes, a/eIF1 favors translation initiation by stabilizing mRNAs that present optimal nucleotide context (Shine-Dalgarno sequence in archaea or Kozak sequence in eukaryotes) helping to locate the initiation codon.

Several structural and biochemical studies have shown that residues of the basic loop of eIF1 were important for the binding of the factor to the ribosome (14,15,17,18,57). Moreover, the cryo-EM structure of a Pa-PIC containing all initiation factors showed that aIF1 was bound to the ribosome in a manner equivalent to its eukaryotic homolog (24). Here, we show that R31, Y32 and K34 of the basic loop of Pa-aIF1 participate in the binding to the 30S. Moreover, some residues, such as Y32, are involved in the stabilization of the 30S:mRNA conformation (Table 1, Supplementary Figure S6). Notably, although the basic character of loop 1 is conserved in archaea and eukaryotes, the consensus sequences in the two phyla are idiosyncratic (Figure 1C). Another notable difference between eukaryotic and archaeal e/aIF2 and their homologues corresponds to the absence of the acidic loop 2 in the archaeal kingdom, as observed here and in (46). In eukaryotes, the acidic loop 2 was shown to participate in start codon selection by interacting with the D-stem-loop of the initiator tRNA (17,58). In addition, many archaeal aIF1 possess a zinc-binding knuckle in their N-terminal domain whereas this is never observed in eukaryotic species. This may reflect adaptation of the role of e/aIF1 to either long-range or local scanning mechanism. Interestingly, variations of the loop 1 and loop 2 sequences were also noted in the SUI1 homologous domains of eIF2D and DENR implicated in eukaryotic re-initiation, another different mechanism for start codon selection (59,60).

**Figure 6.**
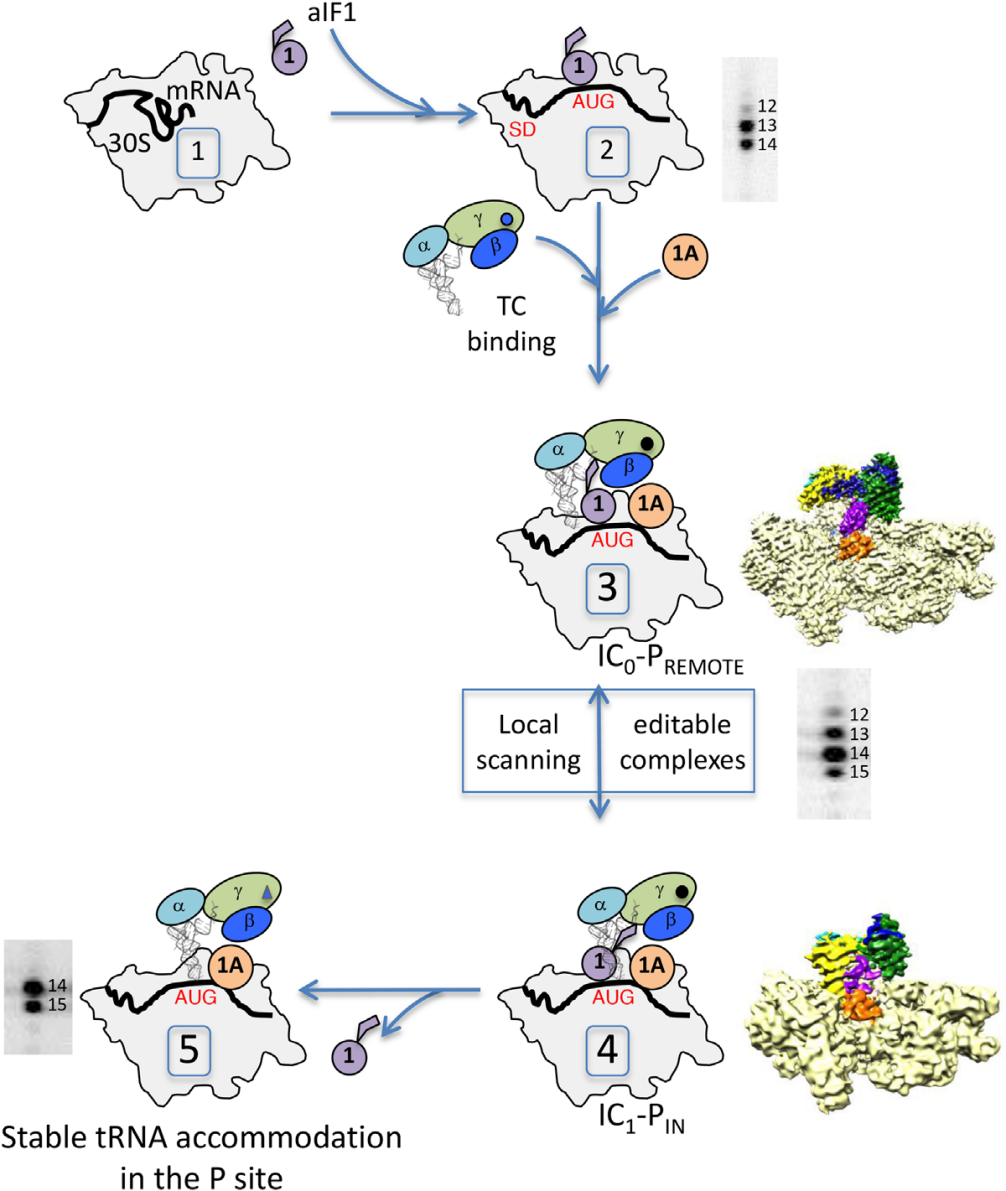
Steps of archaeal translation initiation illustrated by specific toeprinting patterns. Complexes are numbered 1 to 5. The three subunits of aIF2 are represented with ovals. The cryo-EM structures of IC_0_-P_REMOTE_ (complex 3) and IC_1_-P_IN_ (complex 4) are described in (24).

We also used toeprinting experiments to characterize the different states of archaeal translation initiation. Analysis of the toeprinting signal obtained with 30S:mRNA:Met-RNA^Met^ complex defined positions +14 and +15 as those corresponding to the initiator tRNA stably accommodated in the P site. Within the PIC, the toeprint signal is spread out from position +12 to position +15 reflecting a dynamic conformational state with the initiator tRNA not definitely bound in the P site (Figure 6, complexes 3 and 4). The observed dynamic character of the full PIC using toeprinting is consistent with our previous cryo-EM studies identifying two conformational states, IC0-P_REMOTE_ and IC1-P_IN,_ of the ribosomal initiation complex (24). The deduced model was that during translation initiation, the initiator tRNA oscillates between a P_REMOTE_ position to a P_IN_ position, reflecting a local scanning for the search of the start codon. By analogy with the eukaryotic systems (2,10,13), it has been proposed that full initiator tRNA accommodation is obtained after base pairing with the start codon and aIF1 release (24). In the present study, final accommodation of the tRNA is nicely illustrated by the strong toeprint signal at positions +14 and +15 obtained for a PIC complex devoid of aIF1 (Figure 6, complex 5). Therefore, aIF1 is responsible for impairing full tRNA accommodation. In the archaeal cryo-EM structures, aIF1 interacts with the tRNA, the 30S and aIF2 ((24), Figure 6). These interactions may explain the dynamic character of the complex because of partial overlaps of the binding sites of the P site partners, particularly the tRNA and aIF1, as also observed in eukaryotes (17,51). Importantly, the dynamic conformation of the full initiation complex is propitious for proofreading of erroneous initiation complexes, as illustrated in this study by one made with an elongator met-tRNA (Figures 5 and 6). The fidelity function of aIF1, as observed here, is consistent with previous observations using FRET experiments with the *S. solfataricus* system showing that aIF1 specifically destabilized an initiation complex made on a non-AUG mRNA (26). Overall the following model recapitulates the available data for translation of mRNAs having an SD sequence. Initiation factor aIF1 favors the binding and accommodation of the mRNA after SD-anti SD base pairing. The initiator tRNA, inside the TC, scans base triplets in the P site by oscillating between the P_REMOTE_ and P_IN_ positions as defined in (24). This oscillation is favored by the presence of aIF1 that prevents full tRNA accommodation through competition for a same binding site. Base pairing with a proper start codon in the P site ensures a longer stay of the initiator tRNA in the P_IN_ position and thereby favors aIF1 departure that becomes irreversible after full tRNA accommodation. This model also explains how factors other than the sequence of the SD itself can influence the stability of the initiation complex and therefore translation initiation efficiency. Hence, the use of TTG or GTG as start codons will probably lower the level of gene translation, as observed in *E. coli* (61,62), because of less stable base pairing in the P site (63) favoring the P_REMOTE_ conformation. Else, a previous study on 144 genes in *S. solfataricus* has shown that the SD sequence was mainly distributed in a relatively narrow four-base window (50). In our model, a large distance between SD and AUG will increase the dynamics of the mRNA and the structural difference between the P_REMOTE_ and the P_IN_ conformations. As illustrated here by the toeprint results with the Val1Met2-aEF1A-mRNA (Figure 4B), too long a distance between SD and AUG would cause a decrease of translation initiation efficiency.

## DATA AVAILABILITY

The Protein Data Bank accession number for the structure reported in this article is 4MO0.

## Supplementary Material

Supplementary Data
