# Supplementary material for: Role of aIF1 in Pyrococcus abyssi translation initiation
Source: Nucleic Acids Res. 2018 Sep 20;46(20):11061–74. doi: 10.1093/nar/gky850 (PMC6237735; doi:10.1093/nar/gky850)
Supplement: Supplementary Data [file gky850_supplemental_files.pdf]

Role of aIF1 in *Pyrococcus abyssi* translation initiation

Auriane Monestier[1], Christine Lazennec-Schurdevin[1], Pierre-Damien Coureux[1], Yves Mechulam[1] and Emmanuelle Schmitt[1§]

[1]Laboratoire de Biochimie, Ecole polytechnique, CNRS, Université Paris-Saclay, 91128 Palaiseau cedex, France

SUPPLEMENTARY DATA

SUPPLEMENTARY TABLE

	Native	$KPtCl_4$ derivative
**Data collection**		
Space group	$P6_122$	$P6_122$
Cell dimensions		
a, *b*, *c* (Å)	51.4, 51.4, 146.1	50.9, 50.9,147.2
α, β, γ (°)	90.0, 90.0, 120.0	90.0, 90.0, 120.0
Resolution (Å)	32.9-2.1	28.3-2.85
R_{sym}	0.062 (0.578)	0.035 (0.40)
$I / \sigma I$	29.6 (5.5)	18.5 (2.4)
Completeness (%)	99.8 (99.9)	97.3 (100)
Redundancy	18.7 (17.9)	2.85 (2.98)
CC(1/2)	100 (97.4)	99.9 (83.6)
**Refinement**		
Resolution (Å)	47.4–2.1	
No. reflections	7272	
R_{work} / R_{free}	0.220/0.224	
No. atoms		
Protein	627	
Water	38	
B-factors (Å^2)	protein 46<br>water 55	
R.m.s. deviations		
Bond lengths (Å)	0.008	
Bond angles (°)	1.073	

Values in parentheses are for highest-resolution shell.

Table S1: Data collection and refinement statistics for Mj-aIF1 structure determination.

R_{sym} (I)= $\frac{\sum_{hkl}\sum_i |\langle I_{hkl}\rangle - I_{hkl,i}|}{\sum_{hkl}\sum_i |I_{hkl}|}$, where i is the number of reflections hkl.

CC(1/2) is the correlation coefficient between two random half data sets

$$R_{work} = \frac{\sum ||F_{obs}| - |F_{calc}||}{\sum |F_{obs}|}$$

R_{free} is calculated with 5% of the reflections.

Phylum		Number of sequences	% with a putative Zn site
TACK	Candidatus Korarchaeota	4	50
	Candidatus Lokiarchaeota	1	100
	Candidatus Bathyarchaeota	18	100
	Thaumarchaeota	48	90
	Crenarchaeota	78	5
EURYARCHAEOTA	Aciduliprofundum	3	0
	Halobacteria	175	42
	Thermoplasmata	32	59
	Thermococci	29	0
	Theionarchaea	2	100
	Candidatus Altiarchaeales	9	100
	Hadesarchaea	5	100
	Candidatus Thalassoarchaea	2	100
	Methanomicrobia	62	100
	Methanonatronarchaeia	2	100
	Archaeoglobi	8	100
	Methanobacteria	29	100
	Methanococci	16	100
	Methanopyri	1	100
	Others	46	89
DPANN	Nanoarchaeota	3	0
	Candidatus Parvarchaeota	2	100
	Candidatus Aenigmarchaeota	6	100
	Candidatus Diapherotrites	4	100
	Candidatus Micrarchaeota	12	100
	Candidatus Pacearchaeota	16	94
	Candidatus Woesearchaeota	16	81

Table S2: Presence of a putative Zn site in aIF1 sequences from various phyla.

The analysis has been performed on 629 sequences using NCBI taxonomy.

SUPPLEMENTARY FIGURES

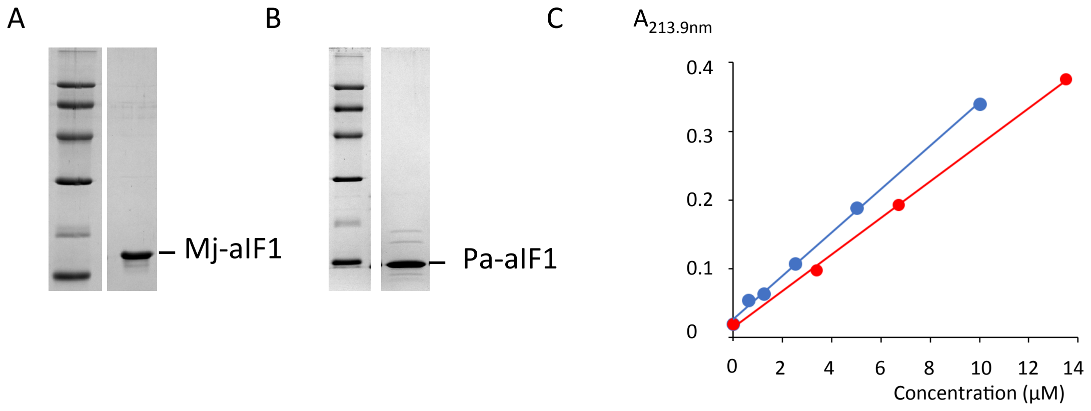

Figure S1: Characterization of aIF1.
A- SDS-PAGE analysis of purified Mj-aIF1 Molecular weight markers: 97, 66, 45, 30, 20.1, 14.4 kDa (GE-Healthcare). B- SDS-PAGE analysis of purified Pa-aIF1. C- Analysis of the zinc content of Mj-aIF1 by atomic absorption spectroscopy. A typical experiment is shown. The graphic shows the absorbance at 213.9 nm from the spectrometer as a function of sample concentration for Mj-aIF1 (red dots) and for a zinc standard (blue dots).

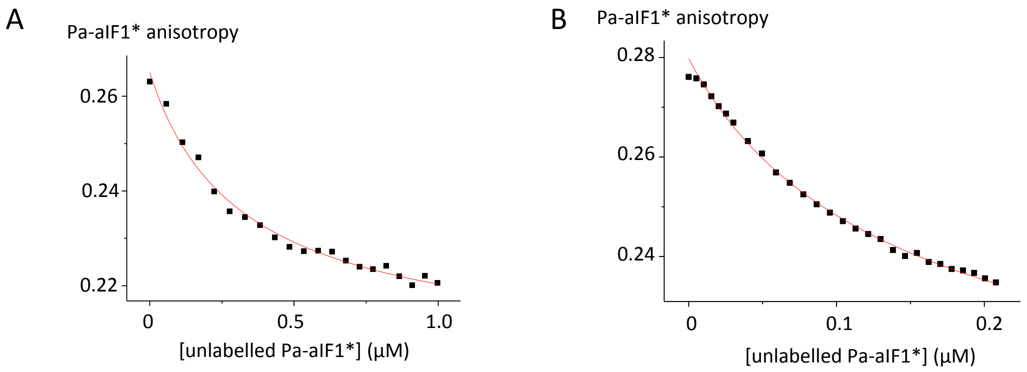

Figure S2: Chase titrations.
A-Chase of coumarin-labeled Pa-aIF1* from its complex with Pa-30S by unlabeled Pa-aIF1*. Initial concentrations were 90 nM (30S) and 15 nM (labeled Pa-aIF1*). Data were fitted as described (Weeks and Crothers, 1992). A Kd of 40 ± 20 nM for unlabeled Pa-aIF1* was derived, in reasonable agreement with the Kd of 12 ± 4 nM measured with the labeled factor.
B-Same experiment but in the presence of 180 nM wt-mRNA. A Kd of 0.4 ± 0.2 nM for unlabeled Pa-aIF1* was derived, in good agreement with the best fit of the data obtained with the labeled factor (Kd of 0.2 nM, Fig. 2).

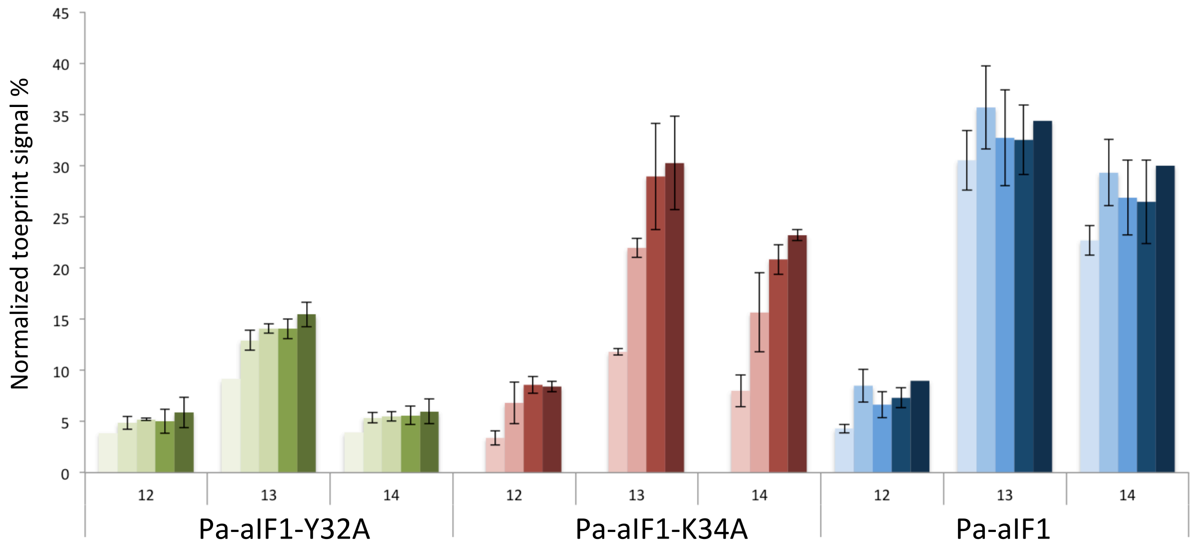

Figure S3: Toeprinting analysis of 30S initiation complexes on wt-aEF1A-mRNA with wild-type and variants of Pa-aIF1.

Normalized toeprint signals obtained with Pa-aIF1-Y32A (green, concentrations were varied from 131.5 nM to 1052 nM from the left to the right), Pa-aIF1-K34A (red, 263 nM to 2104 nM), Pa-aIF1 wt (light blue, from 131.5 to 2104nM). The concentrations of 30S and of mRNA used in all experiments were 25.6 nM and 12.6 nM, respectively. Standard deviations are calculated from at least two independent experiments.

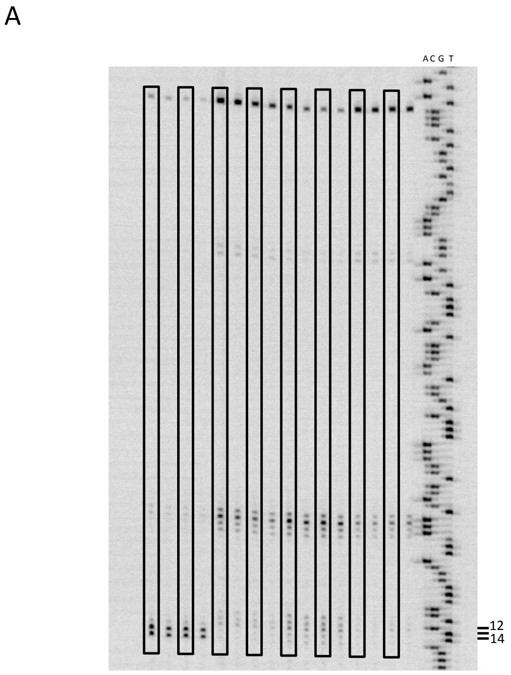

	1	2	3	4	5	6	7	8
wt-mRNA	+	+	-	-	-	-	-	-
mRNA(-7)	-	-	+	+	-	-	-	-
mRNA (-6)	-	-	-	-	+	+	-	-
mRNA (-6/-7)	-	-	-	-	-	-	+	+

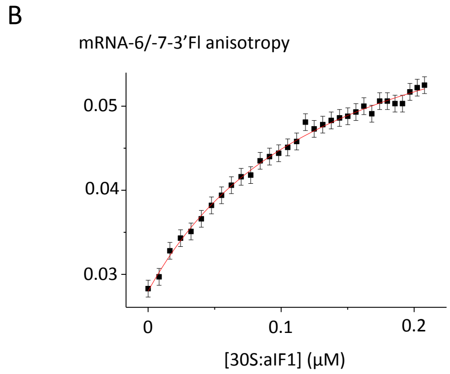

Figure S4: A-Toeprinting analysis of 30S:aIF1 complexes on wt-aEF1A mRNA and on mRNAs mutated at position -6 and -7.

The sequences of the mutated mRNA are shown in Figure 3. The analysis shows that mutations -6 and -7 strongly modify the arrest signal obtained with wt-aEF1A-mRNA at positions +12,+13,+14. The signals observed at upper positions might be due to upstream SD-like sequences. Two concentrations of aIF1 have been tested; 263 nM (black squared boxes) and 5.2 μM (unboxed lanes), corresponding to molar excesses of 10 and 200 respectively, relative to the ribosome concentration.

This experiment shows that positions -6 and -7 participate in the stabilization of the 30S:aIF1 complexes in the vicinity of the start codon.

B-Binding of mRNA-6/-7 3'-labeled with fluorescein to Pa-30S:aIF1 complex. Fluorescence anisotropy of mRNA (10 nM) was followed during titration with Pa-30S:aIF1 and the results were fitted using standard equations for Kd determination. The Kd is deduced from 2 independent experiments was 100 ± 25 nM.

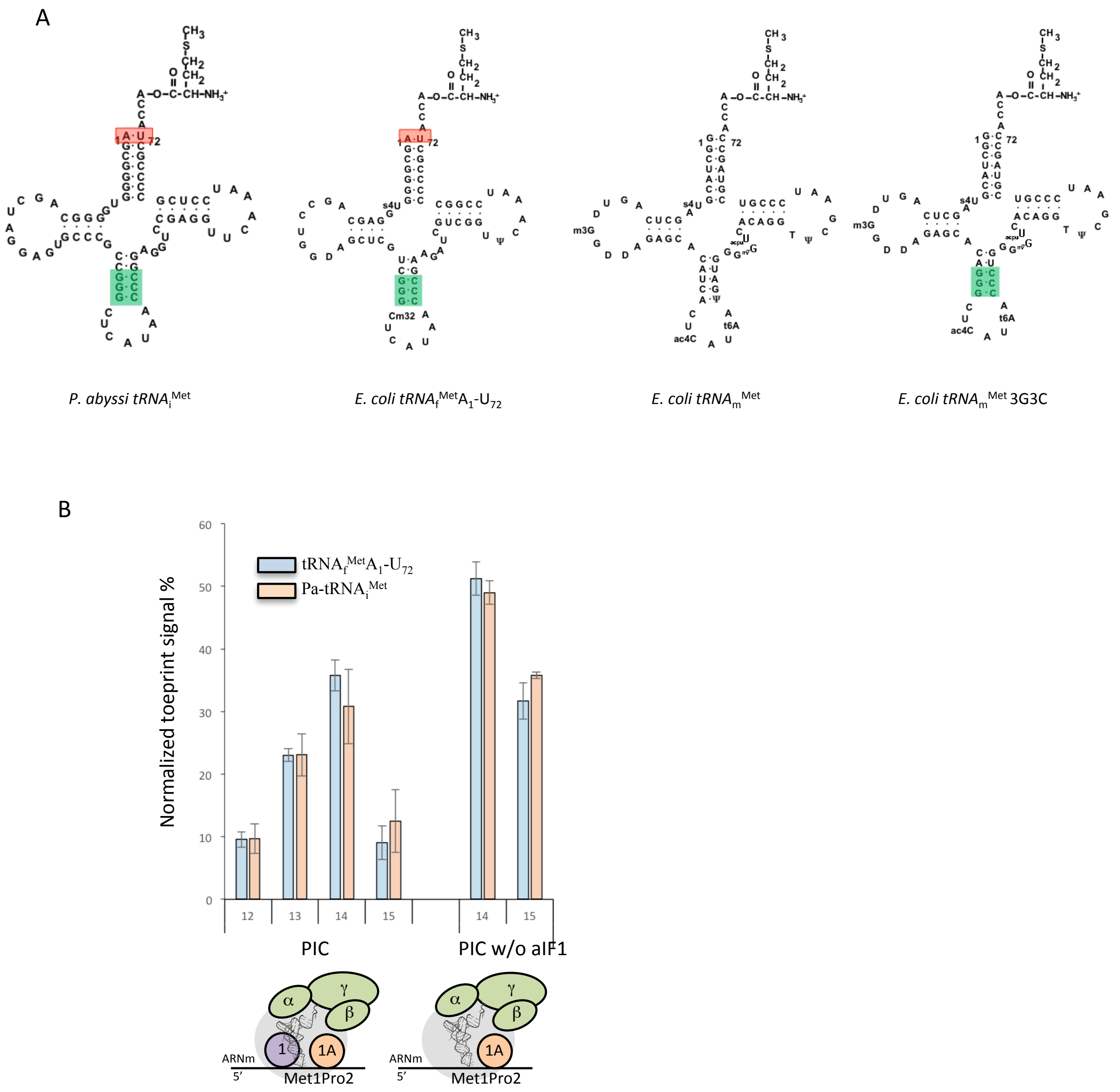

Figure S5: Initiator tRNAs.

A- Cloverleaf representations of Met-tRNAs^{Met}. The A_1-U_{72} base pair at the top of the acceptor stem and the three G-C base pairs in the anticodon stem characteristic of the initiator tRNAs are boxed in red and green, respectively. B- Toeprinting analysis of 30S initiation complexes, as described in Figure 4, assembled on wt-aEF1A-mRNA using Met-tRNA$_f^{Met}$$A_1$-$U_{72}$ or Pa-Met-tRNA$_i^{Met}$. Means and standard deviations are calculated from 5 and 4 independent experiments with Met-tRNA$_f^{Met}$$A_1$-$U_{72}$ or Pa-Met-tRNA$_i^{Met}$, respectively.

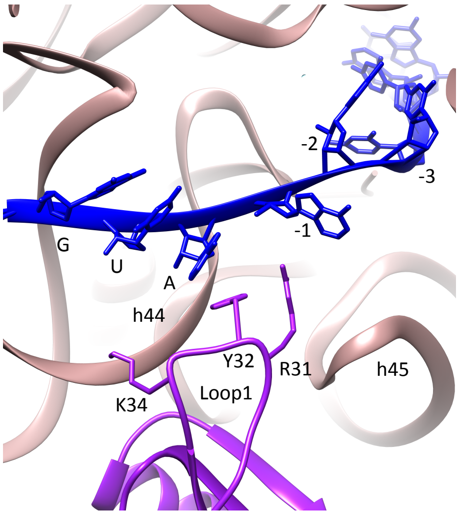

Figure S6: aIF1 binding site on the 30S:mRNA complex.
The view shows the positioning of aIF1 (purple) loop 1 on the 30S:mRNA complex (pink and dark blue, respectively) as observed in the 5.4 Å resolution IC0-P_{REMOTE} structure (2). Notably, conformation of aIF1 loop 1 in 30S:mRNA is different from that observed in free Mj-aIF1.

Supplementary references

1. Weeks, K.M. and Crothers, D.M. (1992) RNA binding assays for Tat-derived peptides: implications for specificity. *Biochemistry*, **31**, 10281-10287.
2. Coureux, P.D., Lazennec-Schurdevin, C., Monestier, A., Larquet, E., Cladiere, L., Klaholz, B.P., Schmitt, E. and Mechulam, Y. (2016) Cryo-EM study of start codon selection during archaeal translation initiation. *Nat Commun*, **7**, 13366.
